# Supplementary material for: The Role of State-of-the-Art Quantum-Chemical Calculations in Astrochemistry: Formation Route and Spectroscopy of Ethanimine as a Paradigmatic Case
Source: Molecules. 2020 Jun 22;25(12):2873. doi: 10.3390/molecules25122873 (PMC7357107; doi:10.3390/molecules25122873)
Supplement: Supplementary file 1 [file molecules-25-02873-s001.pdf]

Article

Supporting Information
The role of state-of-the-art quantum chemical and kinetics calculations in astrochemistry: ethanimine as a paradigmatic case

Carmen Baiano[1], Jacopo Lupi[1], Nicola Tasinato[1], Cristina Puzzarini[2] and Vincenzo Barone[1,*]

1 Scuola Normale Superiore, Piazza dei Cavalieri 7, I-56126 Pisa, Italy
2 Dipartimento di Chimica "Giacomo Ciamician", Università di Bologna, Via F. Selmi 2, 40126 Bologna, Italy
* Correspondence: vincenzo.barone@sns.it

Version June 22, 2020 submitted to Molecules

1. Geometrical parameters

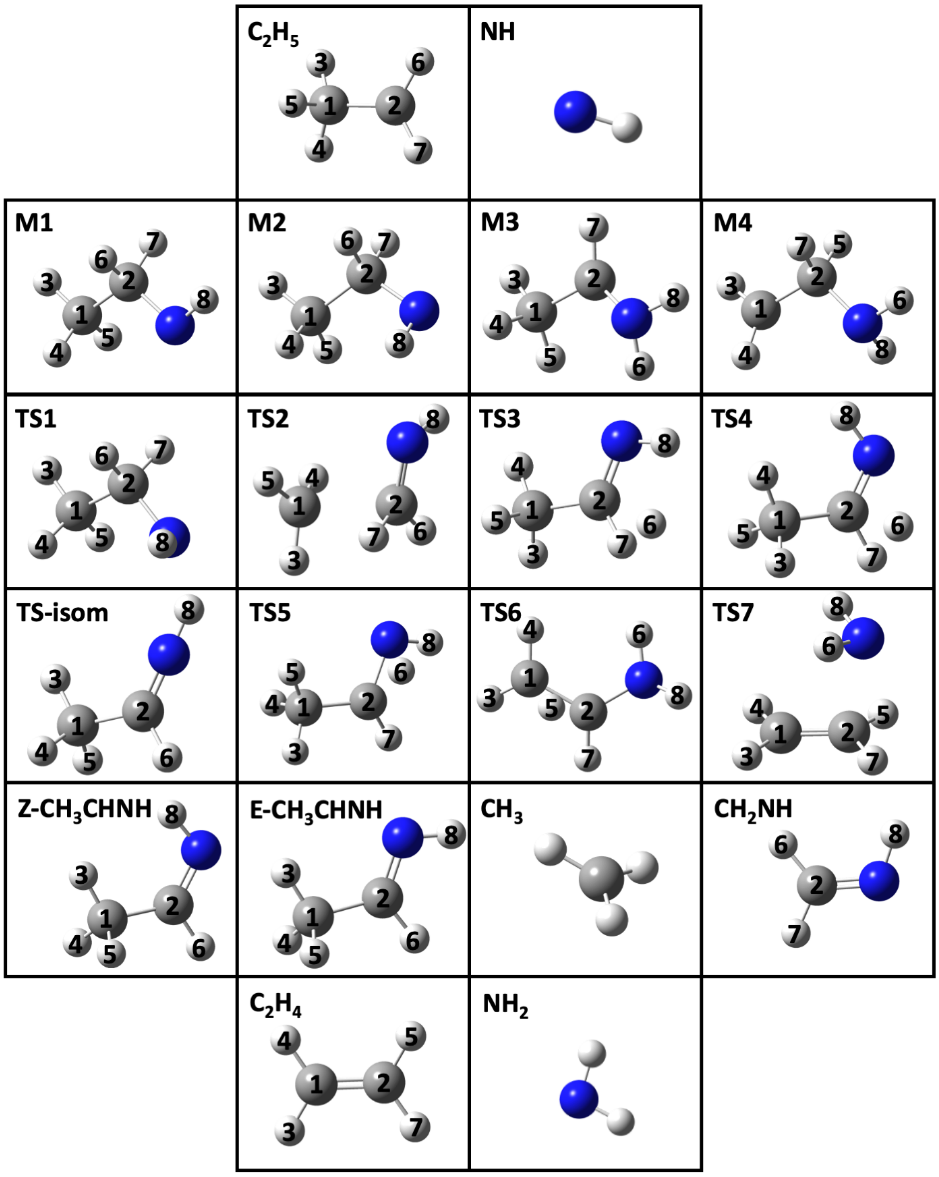

Figure 1. Structures of the species appearing in the formation pathway of ethanimine as obtained at B2PLYP-D3(BJ)/aug-cc-pVTZ level of theory. Numeric labels are used for structural analysis in Table 1

Table 1. Structural parameters of the stationary points of the NH + C_2H_5 reaction at different levels of theory. Distances are in Å, angles in °.

		B3LYP-D3	B2PLYP-D3(BJ)	
		aug-cc-pVTZ	aug-cc-pVTZ	aug-cc-pVQZ
NH	r(NH)	1.040	1.036	1.035
C_2H_5	r(C2H6)	1.080	1.078	1.078
	r(C2H7)	1.080	1.080	1.080
	r(C1C2)	1.484	1.484	1.483
	φ(H6C2C1)	120.9	120.9	120.9
	φ(H3C1C2)	111.9	111.7	111.7
	φ(H3C1H5)	106.3	106.5	106.5
	φ(H5C1H4)	106.3	106.5	106.5
	φ(H6C2H7)	117.7	117.7	117.7
	ϑ(H3C1C2H6)	33.0	33.5	33.4
	ϑ(H5C1C2H7	86.1	85.7	85.8
M1	r(NH8)	1.025	1.023	1.022
	r(C2N)	1.439	1.440	1.439
	r(C1C2)	1.522	1.519	1.518
	r(C1H3)	1.091	1.089	1.089
	r(C1H4)	1.090	1.089	1.088
	r(C1H5)	1.090	1.089	1.088
	r(C2H6)	1.102	1.099	1.099
	r(C2H7)	1.102	1.099	1.099
	φ(C2NH8)	107.6	107.3	107.3
	ϑ(C1C2NH8)	180.0	-180.0	-180.0
	ϑ(H7C2NH8)	57.0	57.2	57.2
	ϑ(H6C2NH8)	-57.0	-57.2	-57.2
M2	r(NH8)	1.026	1.024	1.023
	r(C2N)	1.440	1.441	1.440
	r(C1C2)	1.534	1.530	1.529
	r(C1H3)	1.091	1.089	1.088
	r(C1H4)	1.092	1.090	1.089
	r(C1H5)	1.090	1.089	1.088
	r(C2H6)	1.103	1.100	1.099
	r(C2H7)	1.092	1.090	1.089
	φ(C2NH8)	107.3	106.8	106.9
	ϑ(C1C2NH8)	-46.3	-49.0	-49.1
	ϑ(H7C2NH8)	-170.4	-172.6	-172.7
	ϑ(H6C2NH8)	74.9	71.9	71.8
M3	r(NH8)	1.008	1.008	1.007
	r(NH6)	1.010	1.009	1.008
	r(C2N)	1.398	1.398	1.396
	r(C1C2)	1.487	1.487	1.486
	r(C1H3)	1.089	1.088	1.087
	r(C1H4)	1.101	1.098	1.098
	r(C1H5)	1.096	1.093	1.093
	r(C2H7)	1.082	1.080	1.080
	φ(C2NH6)	115.2	114.7	114.8
	φ(C2NH8)	115.5	115.1	115.3
	ϑ(C1C2NH6)	40.3	41.7	41.4
	ϑ(C1C2NH8)	172.1	172.3	172.4
	ϑ(H7C2NH6)	-170	-170.2	-170.3
	ϑ(H7C2NH8)	-38.1	-39.5	-39.2
M4	r(NH8)	1.013	1.012	1.011
	r(NH6)	1.012	1.011	1.010
	r(C2N)	1.467	1.466	1.464
	r(C1C2)	1.482	1.482	1.481
	r(C1H3)	1.080	1.078	1.078
	r(C1H4)	1.081	1.079	1.078
	r(C2H7)	1.101	1.098	1.098
	r(C2H5)	1.101	1.098	1.097
	φ(C2NH6)	111.1	110.8	110.9
	φ(C2NH8)	110.6	110.3	110.3
	ϑ(C1C2NH6)	-175.8	-176.4	-176.2
	ϑ(C1C2NH8)	64.8	65.0	65.0
	ϑ(H5C2NH6)	59.8	59.6	59.8
	ϑ(H7C2NH6)	-55.9	-56.6	-56.4
	ϑ(H5C2NH8)	-59.5	-59.0	-59.0
	ϑ(H7C2NH8)	-175.3	-175.2	-175.2

Table 1 continued from previous page

TS1	r(NH8)	1.023	1.021	1.021
	r(C2N)	1.445	1.446	1.446
	r(C1C2)	1.539	1.534	1.534
	r(C1H3)	1.091	1.089	1.089
	r(C1H4)	1.090	1.089	1.089
	r(C1H5)	1.089	1.088	1.088
	r(C2H6)	1.095	1.093	1.093
	r(C2H7)	1.097	1.094	1.094
	φ(C2NH8)	109.0	108.9	108.9
	ϑ(C1C2NH8)	-114.2	-114.6	-114.6
	ϑ(H7C2NH8)	129.0	128.6	128.6
	ϑ(H6C2NH8)	9.7	9.1	9.1
TS2	r(NH8)	1.020	1.019	1.019
	r(C2N)	1.293	1.293	1.293
	r(C1C2)	2.271	2.224	2.224
	r(C1H3)	1.081	1.080	1.080
	r(C1H4)	1.080	1.078	1.078
	r(C1H5)	1.079	1.078	1.078
	r(C2H6)	1.091	1.090	1.090
	r(C2H7)	1.087	1.085	1.085
	φ(C2NH8)	110.8	110.5	110.5
	ϑ(C1C2NH8)	-89.8	-89.5	-89.5
	ϑ(H7C2NH8)	172.7	172.7	172.7
	ϑ(H6C2NH8)	10.8	11.0	11.0
TS3	r(NH8)	1.019	1.018	1.018
	r(C2N)	1.284	1.286	1.286
	r(C1C2)	1.501	1.500	1.500
	r(C1H3)	1.090	1.088	1.088
	r(C1H4)	1.088	1.086	1.086
	r(C1H5)	1.094	1.092	1.092
	r(C2H6)	1.926	1.829	1.829
	r(C2H7)	1.096	1.094	1.094
	φ(C2NH8)	111.1	110.7	110.7
	ϑ(C1C2NH8)	175.0	174.2	174.2
	ϑ(H7C2NH8)	7.6	8.3	8.3
	ϑ(H6C2NH8)	-79.5	-80.8	-80.8
TS4	r(NH8)	1.022	1.021	1.021
	r(C2N)	1.283	1.285	1.285
	r(C1C2)	1.508	1.506	1.506
	r(C1H3)	1.090	1.088	1.088
	r(C1H4)	1.093	1.092	1.092
	r(C1H5)	1.090	1.089	1.089
	r(C2H6)	1.091	1.090	1.090
	r(C2H7)	1.927	1.830	1.830
	φ(C2NH8)	110.9	110.4	110.4
	ϑ(C1C2NH8)	8.7	9.6	9.6
	ϑ(H7C2NH8)	-97.2	-96.4	-96.4
	ϑ(H6C2NH8)	175.7	175.2	175.2
TS-isom	r(NH8)	0.987	0.987	0.987
	r(C2N)	1.234	1.237	1.237
	r(C1C2)	1.512	1.510	1.510
	r(C1H3)	1.088	1.087	1.087
	r(C1H4)	1.094	1.092	1.092
	r(C1H5)	1.094	1.092	1.092
	r(C2H6)	1.110	1.107	1.107
	φ(C2NH8)	179.4	179.4	179.4
	ϑ(C1C2NH8)	-0.1	0.6	0.6
	ϑ(H6C2NH8)	179.9	-179.4	-179.4
TS5	r(NH8)	1.020	1.020	1.019
	r(NH6)	1.252	1.251	1.251
	r(C2N)	1.452	1.453	1.451
	r(C1C2)	1.494	1.492	1.491
	r(C1H3)	1.090	1.089	1.088
	r(C1H4)	1.097	1.095	1.094
	r(C1H5)	1.091	1.089	1.088
	r(C2H6)	1.284	1.276	1.275
	r(C2H7)	1.087	1.085	1.084
	φ(C2NH8)	107.6	107.2	107.3
	ϑ(C1C2NH8)	-165.1	-165.6	-165.6
	ϑ(H7C2NH8)	-14.0	-14.4	-14.4

Table 1 continued from previous page

	ϑ(H7C2NH6)	-106.2	-106.3	-106.3
TS6	r(NH8)	1.009	1.009	1.008
	r(NH6)	1.009	1.009	1.008
	r(C2N)	1.413	1.413	1.411
	r(C1C2)	1.483	1.481	1.480
	r(C1H3)	1.080	1.078	1.077
	r(C1H4)	1.080	1.079	1.078
	r(C1H5)	1.326	1.319	1.318
	r(C2H5)	1.306	1.300	1.299
	r(C2H7)	1.083	1.081	1.080
	φ(C2NH8)	114.7	114.4	114.5
	φ(C2NH6)	113.9	113.3	113.4
	ϑ(C1C2NH8)	145.5	144.1	144.1
	ϑ(C1C2NH6)	16.8	16.6	16.3
	ϑ(H7C2NH8)	-66.2	-67.8	-67.6
	ϑ(H7C2NH6)	165.1	164.8	164.6
TS7	r(NH8)	1.024	1.023	1.022
	r(NH6)	1.024	1.023	1.022
	r(C2N)	2.184	2.156	2.154
	r(C1C2)	1.353	1.351	1.350
	r(C1H3)	1.082	1.080	1.080
	r(C1H4)	1.082	1.080	1.080
	r(C2H5)	1.080	1.079	1.079
	r(C2H7)	1.080	1.079	1-079
	φ(C2NH8)	98.6	98.6	98.6
	φ(C2NH6)	98.6	98.6	98.6
	ϑ(C1C2NH8)	-52.5	-52.4	-52.4
	ϑ(C1C2NH6)	52.5	52.4	52.4
	ϑ(H7C2NH8)	-174.6	-174.5	-174.5
	ϑ(H7C2NH6)	-69.7	-69.6	-69.6
CH_3	r(CH)	1.078x3	1.076x3	1.075x3
	φ(HCH)	120.0x3	120.0x3	120.0x3
CH_2NH	r(C2N)	1.264	1.269	1.268
	φ(C2NH8)	111.4	110.9	110.9
	φ(H6C2H7)	116.1	116.7	116.7
	ϑ(H6C2NH8)	0.0	0.0	0.0
	ϑ(H7C2NH8)	180.0	180.0	180.0
E-CH_3CHNH	r(C2N)	1.267	1.272	1.270
	r(C1C2)	1.495	1.493	1.492
	r(C2H6)	1.097	1.094	1.094
	φ(C2NH8)	111.5	110.8	110.9
	ϑ(C1C2NH8)	180.0	180.0	180.0
	ϑ(H6C2NH8)	0.0	0.0	0.0
Z-CH_3CHNH	r(C2N)	1.266	1.271	1.269
	r(C1C2)	1.500	1.498	1.498
	r(C2H6)	1.093	1.090	1.090
	φ(C2NH8)	111.3	110.6	110.6
	ϑ(C1C2NH8)	0.0	0.0	0.0
	ϑ(H6C2NH8)	180.0	180.0	180.0
NH_2	r(NH)	1.027	1.024	1.023
	φ(HNH)	103.3	103.3	103.4
C_2H_4	r(C1C2)	1.325	1.328	1.327
	φ(HCC)	121.7x3	121.6x3	121.6x3
	φ(HCH)	116.5x2	116.9x2	116.9x2
	ϑ(H4C1C2H5)	0.0	0.0	0.0
	ϑ(H4C1C2H7)	180.0	180.0	180.0

2. Absolute energies

Table 2. Energies are in E_h. Reference geometries at B2PLYP-D3(BJ)/aug-cc-pVTZ level of theory.

	B2PLYP-D3(BJ)/aug-cc-pVTZ	CCSD(T)/CBS+CV		CCSD(T)/CBS+CV+fT+pQ		ChS
		T,Q	Q,5	T,Q	Q,5	
NH	-55.2025220	-55.2143895	-55.2190340	-55.2149417	-55.2195863	-55.2141825
C_2H_5	-79.1066887	-79.1401015	-79.1476187	-79.1411415	-79.1486587	-79.1405321
M1	-134.4406761	-134.4886002	-134.5007278	-134.4901229	-134.5022506	-134.4890983
M2	-134.4395633	-134.4875802	-134.4997073	-134.4891120	-134.5012390	-134.4881290
M3	-134.4550505	-134.5026174	-134.5147721	-	-	-134.5033022
M4	-134.4374715	-134.4862685	-134.4983261	-	-	-134.4870289
TS1	-134.4379875	-134.4859987	-134.4981322	-134.4875296	-134.4996631	-134.4865507
TS2	-134.3944707	-134.4406919	-134.4529517	-134.4433467	-134.4556065	-134.4413882
TS3	-134.3836749	-134.4314920	-134.4437482	-134.4337551	-134.4460113	-134.4319856
TS4	-134.3826904	-134.4305599	-134.4428110	-134.4328182	-134.4450693	-134.4310511
TS-isom	-133.8465558	-133.8919738	-133.9042667	-133.8934507	-133.9057435	-133.8929897
TS5	-134.3817405	-134.4296502	-134.4417896	-	-	-134.4305127
TS6	-134.3764047	-134.4239981	-134.4361661	-	-	-134.4249503
TS7	-134.3991008	-134.4450773	-134.4574038	-	-	-134.4456568
CH_2NH	-94.5902902	-94.6208211	-94.6293610	-94.6219034	-94.6304432	-94.6216334
CH_3	-39.8125589	-39.8293382	-39.8330384	-39.8299194	-39.8336195	-39.8297072
E-CH_3CHNH	-133.8919820	-133.9391388	-133.9514338	-133.9406117	-133.9529067	-133.9400468
Z-CH_3CHNH	-133.8908845	-133.9380799	-133.9503673	-133.9395581	-133.9518454	-133.9389855
H	-0.4986682	-0.5000222	-0.5000222	-0.5000222	-0.5000222	-0.5000222
C_2H_4	-78.5427309	-78.5759224	-78.5836340	-	-	-78.5765986
NH_2	-55.8589088	-55.8732924	-55.8777758	-	-	-55.8736928

3. $\mathcal{T}_1$ diagnostic

Table 3. Coupled-cluster $\mathcal{T}_1$ diagnostic for all the species considered.

	$\mathcal{T}_1$	
	cc-pVTZ	cc-pVQZ
NH	0.010	0.011
C_2H_5	0.010	0.010
M1	0.012	0.013
M2	0.012	0.013
M3	0.014	0.014
M4	0.010	0.011
TS1	0.012	0.013
TS2	0.031	0.031
TS3	0.031	0.031
TS4	0.031	0.031
TS-isom	0.011	0.012
TS5	0.016	0.016
TS6	0.016	0.016
TS7	0.031	0.031
CH_2NH	0.012	0.012
CH_3	0.008	0.009
E-CH_3CHNH	0.012	0.012
Z-CH_3CHNH	0.012	0.012
C_2H_4	0.011	0.011
NH_2	0.008	0.009

4. CCSD(T)/CBS+CV+fT+pQ kinetic results

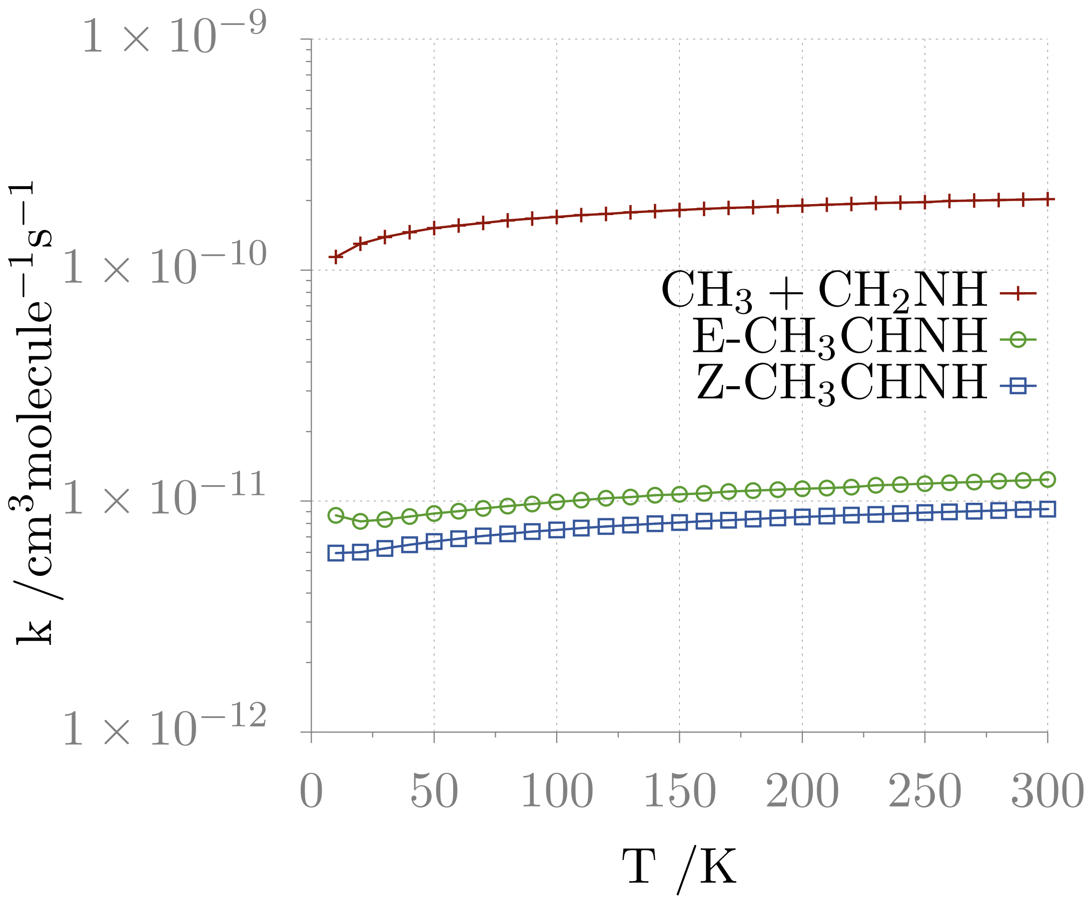

Figure 2. Rate coefficients as a function of temperature.

© 2020 by the authors. Submitted to *Molecules* for possible open access publication under the terms and conditions of the Creative Commons Attribution (CC BY) license (http://creativecommons.org/licenses/by/4.0/).

Table 4. Product-formation rate constants (in cm^3 $molecule^{-1}$ s^{-1}) at 1×10^{-12} atm as a function of the temperature.

T(K)	$CH_3 + CH_2NH$	E-CH_3CHNH	Z-CH_3CHNH
10	1.14×10^{-10}	8.68×10^{-12}	5.96×10^{-12}
20	1.30×10^{-10}	8.17×10^{-12}	6.01×10^{-12}
30	1.39×10^{-10}	8.33×10^{-12}	6.24×10^{-12}
40	1.46×10^{-10}	8.58×10^{-12}	6.48×10^{-12}
50	1.52×10^{-10}	8.83×10^{-12}	6.69×10^{-12}
60	1.56×10^{-10}	9.05×10^{-12}	6.87×10^{-12}
70	1.60×10^{-10}	9.30×10^{-12}	7.06×10^{-12}
80	1.64×10^{-10}	9.52×10^{-12}	7.22×10^{-12}
90	1.67×10^{-10}	9.72×10^{-12}	7.38×10^{-12}
100	1.70×10^{-10}	9.91×10^{-12}	7.51×10^{-12}
110	1.73×10^{-10}	1.01×10^{-11}	7.64×10^{-12}
120	1.75×10^{-10}	1.03×10^{-11}	7.76×10^{-12}
130	1.78×10^{-10}	1.04×10^{-11}	7.88×10^{-12}
140	1.80×10^{-10}	1.06×10^{-11}	7.98×10^{-12}
150	1.82×10^{-10}	1.07×10^{-11}	8.08×10^{-12}
160	1.84×10^{-10}	1.08×10^{-11}	8.18×10^{-12}
170	1.86×10^{-10}	1.10×10^{-11}	8.27×10^{-12}
180	1.87×10^{-10}	1.11×10^{-11}	8.36×10^{-12}
190	1.89×10^{-10}	1.12×10^{-11}	8.45×10^{-12}
200	1.90×10^{-10}	1.13×10^{-11}	8.53×10^{-12}
210	1.92×10^{-10}	1.14×10^{-11}	8.61×10^{-12}
220	1.93×10^{-10}	1.15×10^{-11}	8.69×10^{-12}
230	1.95×10^{-10}	1.17×10^{-11}	8.76×10^{-12}
240	1.96×10^{-10}	1.18×10^{-11}	8.84×10^{-12}
250	1.97×10^{-10}	1.19×10^{-11}	8.91×10^{-12}
260	1.99×10^{-10}	1.20×10^{-11}	8.98×10^{-12}
270	2.00×10^{-10}	1.21×10^{-11}	9.05×10^{-12}
280	2.01×10^{-10}	1.22×10^{-11}	9.11×10^{-12}
290	2.02×10^{-10}	1.23×10^{-11}	9.18×10^{-12}
300	2.03×10^{-10}	1.24×10^{-11}	9.24×10^{-12}

Table 5. Product branching ratios at various temperatures.

Branching ratios	$CH_3 + CH_2NH$	E-CH_3CHNH + H	Z-CH_3CHNH + H
10 K	88.6 %	6.8 %	4.6%
100 K	90.7 %	5.3 %	4.0 %
300 K	90.4 %	5.5 %	4.1 %
